# The impact of brain cancer care coordinators on healthcare utilization and outcomes in patients with glioblastoma

**DOI:** 10.1093/nop/npae030

**Published:** 2024-05-04

**Authors:** Martin Hong, Lucy Leigh, Connor Ballinger, Penny Reeves, Alisha Gooley, Sandy Nixon, Chris Paul, James Lynam

**Affiliations:** Department of Medical Oncolgy, Liverpool Hospital, Liverpool, New South Wales, Australia; School of Medicine, Western Sydney University, Sydney, New South Wales, Australia; Data Sciences, Hunter Medical Research Institute, New Lambton Heights, New South Wales, Australia; Health Economics, Hunter Medical Research Institute, New Lambton Heights, New South Wales, Australia; Health Economics, Hunter Medical Research Institute, New Lambton Heights, New South Wales, Australia; School of Medicine and Public Health, University of Newcastle, Callaghan, New South Wales, Australia; Department of Neurosurgery, John Hunter Hospital, New Lambton Heights, New South Wales, Australia; Mark Hughes Foundation, Hamilton, New South Wales, Australia; Department of Neurosurgery, John Hunter Hospital, New Lambton Heights, New South Wales, Australia; Mark Hughes Foundation, Hamilton, New South Wales, Australia; School of Medicine and Public Health, University of Newcastle, Callaghan, New South Wales, Australia; Department of Medical Oncolgy, Calvary Mater Newcastle, Waratah, New South Wales, Australia; School of Medicine and Public Health, University of Newcastle, Callaghan, New South Wales, Australia

**Keywords:** care pathway, glioblastoma patients, glioblastoma, health economics, supportive care

## Abstract

**Background:**

Cancer care coordinators (CCCs) are recognized as having an important role in patients’ and carers’ cancer journey. However, there are no studies investigating the impact of CCCs on quantitative outcomes. We performed a retrospective cohort study investigating the impact of brain cancer care coordinators (BCCCs) on health service resource use and survival in patients with glioblastoma.

**Methods:**

All patients diagnosed with glioblastoma between 2012 and 2019 in Hunter New England Local Health District, Australia (HNELHD) were included and the patients were divided into 2 cohorts: before and after the introduction of the BCCC. Any patient diagnosed in 2016, during the introduction of the BCCC, were excluded. The main outcomes assessed were overall survival, health service resource use, odds of being admitted to hospital after the emergency presentation, and cost-offset analysis to examine the economic implications of BCCCs.

**Results:**

A total of 187 patients were included. There were no significant differences in overall survival between the 2 groups (mOS 12.0 vs 11.16 months, HR 0.95). However, there was a reduction in the number of ED presentations and admissions. This was associated with a 24% reduction in aggregate length of stay with the BCCC. There was no statistically significant difference in mean patient costs, however our hospital may have saved over AUD$500 000 with BCCCs.

**Conclusions:**

The introduction of BCCC did not improve survival but appeared to be associated with reduced health resource utilization. This study provides economic justification, in addition to the established quality of life improvements, to support the presence of BCCCs.

The Australian Institute of Health and Welfare (AIHW) predicted that in 2021 there would be approximately 151 000 new cases of cancer diagnosed, and cancer accounted for about 1 in 9 hospitalizations (1 328 838 hospitalizations in total) between 2019 and 2020.^1^ A separate report on central nervous system (CNS) cancers estimated that approximately 1908 new cases of CNS cancers will be diagnosed in 2017.^2^ Notably, 62% of all CNS cancers diagnosed in 2013 were glioblastoma multiforme (GBM) and which has a poor median survival, ranging between 14 and 16 months^2–4^

The current mainstay of management of glioblastoma (GBM) is the Stupp protocol, which involves surgical resection, followed by concurrent chemotherapy–radiotherapy for 6 weeks with temozolomide, then with temozolomide alone for another 6 monthly cycles at a higher dose.^3^ The coordination of the treatment according to the regime requires close communication between multiple medical departments: Neurosurgery, Medical Oncology, Radiation Oncology, Palliative Care and Allied Health Departments, and for the patient to negotiate the complex healthcare system. Unfortunately, following progression there is no recognized standard regimen.^5^

In multiple oncological areas, the importance of cancer care coordination is thought to be critical in the delivery of high quality healthcare, especially with respect to the patient’s experience with healthcare, and the quality of end-of-life care.^6^ Time and again, multiple studies have shown that patient navigators or cancer care coordinators improve access to care, adherence to therapy, and improve patient satisfaction around their treatment and care.^7–10^ However there is a gap in knowledge on how cancer care coordination may impact on quantitative outcomes for patients, such as survival, number of hospital presentations, or length of hospital stay, which directly affect the patient’s cancer journey. A previous study which investigated the cost-utility of cancer care coordinators in Stage III colon cancers performed by a group in New Zealand relied on modeling based on estimations of factors which a cancer coordinator would affect in a patient’s care, and showed that the use of cancer care coordinators in colon cancer management was cost-effective, and also improved health outcomes for some groups, such as the younger patients and Maori patients.^11^ Furthermore, a 2010 study from the US showed that in patients with non–small cell lung cancer, those who were randomized to early involvement of palliative care services (palliative care physicians and specialist nurses) not only had better quality of life than those who had palliative care involvement at the discretion of their oncologist, but also longer survival.^12^

In the Hunter New England Local Health District (HNELHD), with funding from the Mark Hughes Foundation, the role of the brain cancer care coordinator (BCCC) was set up in 2016 to provide comprehensive support of patients with diagnosis of primary brain tumors and their families through diagnosis, treatment, clinical trials, symptom management, follow-up in the community and end of life care. The BCCC are specialized nurses involved with all patients diagnosed with primary brain cancers in the HNELHD and assist with not only navigation, but also with any symptom and emotional support for patients and carers during their treatments. These specialized clinicians provide assistance not only in-person at the hospitals but also via phone calls.

This study set out to investigate the impact of the BCCC on quantitative health utilization measures and patient outcomes in the management of people with glioblastoma multiforme.

## Methods

### Study Design

We performed a retrospective cohort study of all patients aged ≥18 diagnosed with GBM in the HNE Local Health District (HNELHD) between October 2012 and December 2019. All patients were identified from the medical records of patients referred to the Medical Oncology and Radiation Oncology departments in HNELHD and to the Medical Oncology department at Newcastle Private Hospital using the C71 ICD-10 codes with subsequent manual filtering for GBM patients only. Patients were divided into 2 groups: patients diagnosed prior to the introduction of the BCCC in 2016 (pre-BCCC) and those who were diagnosed after the introduction of the BCCC in 2017 (post-BCCC). For those in the post-BCCC group, they were further stratified into patients who had contact with the BCCC (BCCC contact) and those who did not (No contact). Contact was defined as a documented entry by the BCCC in the patients Electronic Medical Record (EMR).

The BCCC was first introduced in 2016 with a pilot year, thus all patients diagnosed in 2016 were excluded from this study.

### Data Collection

All data were collected from the EMR used within the HNELHD (Clinical Applications Portal, Orion Health; ARIA, Varian Medical Systems) and Newcastle Private Hospital (CHARM, Citadel Health). For health economics analysis, only patient data from HNELHD were used.

Patient data were collected and managed using REDCap electronic data capture tools hosted at the Calvary Mater Newcastle.^13,14^

Health service resource use data were collected based on the diagnosis-related groups (DRGs) for hospital admissions and urgency-related groups (URGs) and urgency disposition groups (UDGs) for emergency department presentations from the public hospital EMR. Data linkage methods were employed to identify and measure inpatient and emergency admission events. For the purpose of event valuation, price weights reflecting the cost of each admission were also extracted.

Care types of admissions were reviewed using the care type definitions developed by the AIHW, last revised in 2019.^15^ Acute care is defined as care in which the primary clinical purpose or treatment goal is to manage labor (obstetric), cure illness or provide definitive treatment of injury, perform surgery, relieve symptoms of illness or injury (excluding palliative care), reduce severity of an illness or injury, protect against exacerbation and/or complication of an illness and/or injury, which could threaten life or normal function, or perform diagnostic or therapeutic procedures. Palliative care is defined as care in which the primary clinical purpose or treatment goal is an optimization of the quality of life of a patient with an active and advanced life-limiting illness. The patient will have complex physical, psychosocial, and/or spiritual needs. Mixed care was defined as an admission which contained both care types during the admission.

Health service resource data was unable to be obtained from private institutions. While conceivably some patients treated through the private institutions may have used said institutions, general practice within HNELHD would be that acutely unwell patients would either present to the public neurosurgical institution or the tertiary care oncological institution, both of which are public.

### Cost-Offset Analysis

The cost-offset analysis compared the BCCC investment against the valuation of any health care resource use difference between the pre and post cohorts. Under the assumption that there would be negligible differences in routine resource use between the 2 periods as treatment approaches had remained constant, but there may be differences in ad hoc hospital interactions, the economic analysis was constrained to examine emergency department presentations and any associated admissions, ignoring planned hospital interactions.

The analysis was performed following The Independent Health and Aged Care Pricing Authority (IHACPA) guidelines in estimating cost for treating patients in a public hospital.^7^ Australian Refined Diagnosis Related Groups (DRGs) were used to classify the resources used by patient’s during their ED and hospital admissions during the study period. This was then used to derive the Nationally Weighted Activity Unit (NWAU), which can then be multiplied by the National Efficient Price (NEP) to estimate the cost of a patient’s treatment. The IHACPA publishes the NEP each financial year, and using the total health price index published by The Australian Institute of Health and Welfare (AIHW),^8^ the NEPs were adjusted to 2019–2020 prices to ensure an accurate comparison of the costs incurred during the study period.

### Statistical Plan

Descriptive statistics for patient characteristics were presented as count (%), or mean (SD) and median (min, max), if continuous. *P*-values were generated by Chi-Square Test of Independence for distributions of categorical data, and ANOVA and Kruskal–Wallis for parametric or nonparametric distributions of continuous data.

All patient survival statistical analyses were programmed using R v4.2.0 (R Foundation for Statistical Computing, Vienna, Austria). A priori, *P* < .05 (2-tailed) was used to indicate statistical significance. Patient survival was estimated using the Kaplan–Meier method and patients who were diagnosed in the pre-BCCC period and who survived beyond the pre-BCCC period had their survival data truncated to December 31, 2015 and censored to account for any impact the introduction of the BCCC may have had. Truncation and censoring were also applied to post-BCCC period diagnosed patients surviving past December 31, 2019. Both univariate and multivariate Cox proportional hazards models were used to estimate the effect of the BCCC and known prognosticating variables on overall survival. The assumption of proportional hazards was tested using the log-rank test.

Patient emergency presentation and length of stay analysis were performed using the R language. Length of stay (LOS) was calculated by summing the total time spent by a patient in Acute Care service category, taking into account contiguous acute care services. This was done also for patients admitted under the Palliative care service category.

### Ethics

The study was approved by the Research Ethics and Governance department at Hunter New England Local Health District (AU202008-13, AU20222-01).

## Results

### Patient Characteristics

Overall, 199 patients who had a diagnosis of glioblastoma between 2013 and 2019 were identified in the hospital databases. 12 patients were excluded from the analysis (10 patients were diagnosed in 2016 during the pilot period and 2 patients did not have glioblastoma on review of the histopathology), leading to a final total of 187 patients included in the final analysis. Approximately 70% of the patients were male, and the mean age at diagnosis was 64 years. As seen in Table 1, the pre-BCCC and post-BCCC groups were well balanced, with the only significant differences being a greater proportion of patients in the post-BCCC group receiving no treatment and a greater proportion receiving chemotherapy through private institutions. Overall, approximately 19% of all patients in this study received part or all of their treatment at a private hospital.

**Table 1.** Baseline Characteristics

	BCCC present		
Characteristic	Overall, *N* = 187^1^	Pre-BCCC, *N* = 91^1^	Post-BCCC, *N* = 96^1^
Age at diagnosis, Mean (*SD)*	64 (11)	64 (11)	64 (11)
Gender, *n* (%)			
Female	63 (34)	33 (36)	30 (31)
Male	124 (66)	58 (64)	66 (69)
Weight, Mean (*SD*)	84 (19)	82 (21)	85 (17)
Location of treatment, *n* (%)			
No treatment	29 (16)	9 (9.9)	20 (21)
Private	19 (10)	2 (2.2)	17 (18)
Private—MO only	2 (1.1)	1 (1.1)	1 (1.0)
Private—RT only	2 (1.1)	1 (1.1)	1 (1.0)
Private MO + Public RT	13 (7.0)	3 (3.3)	10 (10)
Public—Both	99 (53)	60 (66)	39 (41)
Public—RT only	23 (12)	15 (16)	8 (8.3)
MGMT promoter methylation status, *n* (%)			
Promoter methylated	21 (11)	10 (11)	11 (12)
Promoter unmethylated	32 (17)	14 (16)	18 (19)
Untested	132 (71)	66 (73)	66 (69)
IDH1 mutation status, *n* (%)			
IDH mutant	4 (2.1)	2 (2.2)	2 (2.1)
IDH wild type	150 (80)	62 (68)	88 (92)
Untested	33 (18)	27 (30)	6 (6.3)
Extent of surgery, *n* (%)			
Biopsy	23 (12)	11 (12)	12 (13)
Gross macroscopic	120 (64)	62 (68)	58 (60)
Sub-total	38 (20)	13 (14)	25 (26)
Unknown	6 (3.2)	5 (5.5)	1 (1.0)
Had adjuvant TMZ, *n* (%)	113 (60)	56 (62)	57 (59)
Had subsequent systemic treatment after adjuvant therapy, *n* (%)	56 (30)	26 (29)	30 (31)
Number of subsequent systemic treatments, Mean (Range)	1 (1–4)	2 (1–4)	1 (1–3)
Patient status at the time of data collection, *n* (%)			
Alive	17 (9.1)	4 (4.4)	13 (14)
Dead	159 (85)	84 (92)	75 (78)
Lost to follow-up	11 (5.9)	3 (3.3)	8 (8.3)

^1^Mean (*SD*); *n* (%); Mean (Range) as per row.

### Overall Survival

**Figure 1.**
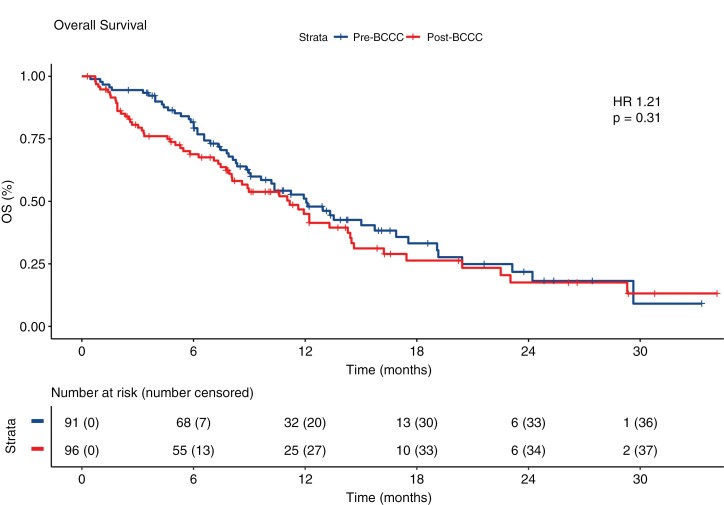
Overall survival.

In the 187 patients analyzed in this study, the median overall survival was 12.0 months (95% confidence interval [CI], 9.0–15.72) prior to the introduction of the BCCC, and 11.16 months (95% CI, 7.92–14.28) after the introduction of the BCCC. There were no significant differences in the overall survival in patients with GBM treated in the HNELHD with or without the BCCC, with an unadjusted hazard ratio of 1.21 (95% CI, 0.84–1.76) (Figure 1). The presence of the BCCC had no effect on overall survival using the Cox proportional hazards model. However, having a methylated MGMT promoter and having commenced concurrent chemoradiotherapy as per the Stupp protocol all had statistically significant independent benefit on survival which correlate with known factors in literature which have positive impact on survival (Table 2).

**Table 2.** Cox Proportional Hazards Analysis for OS

	Univariate				Multivariate			
Characteristic	*N*	Event *N*	HR (95% CI)^1^	*P*-value	*N*	Event *N*	HR (95% CI)^*1*^	*P*-value
BCCC present								
Pre-BCCC	91	54	–		90	53	–	
Post-BCCC	96	57	1.21 (0.84–1.76)	.31	95	56	1.36 (0.92–2.01)	.12
Gender								
Male	124	81	–		122	79	–	
Female	63	30	0.70 (0.46–1.07)	.10	63	30	0.58 (0.38–0.90)	.015
Age at diagnosis	187	111	1.03 (1.01–1.05)	.003	185	109	1.02 (1.00–1.04)	.074
MGMT promoter methylation status								
Promoter unmethylated	32	25	–		32	25	–	
Promoter methylated	21	14	0.43 (0.22–0.83)	.012	21	14	0.47 (0.24–0.93)	.030
Untested	132	70	0.68 (0.43–1.08)	.10	132	70	1.01 (0.62–1.63)	.97
Had concurrent RT + TMZ								
Yes	134	68	–		132	66	–	
No	53	43	4.85 (3.20–7.35)	<.001	53	43	5.39 (3.42–8.48)	<.001

^1^HR = Hazard Ratio, CI = Confidence Interval.

### Hospital Presentations and Admissions

**Figure 2.**
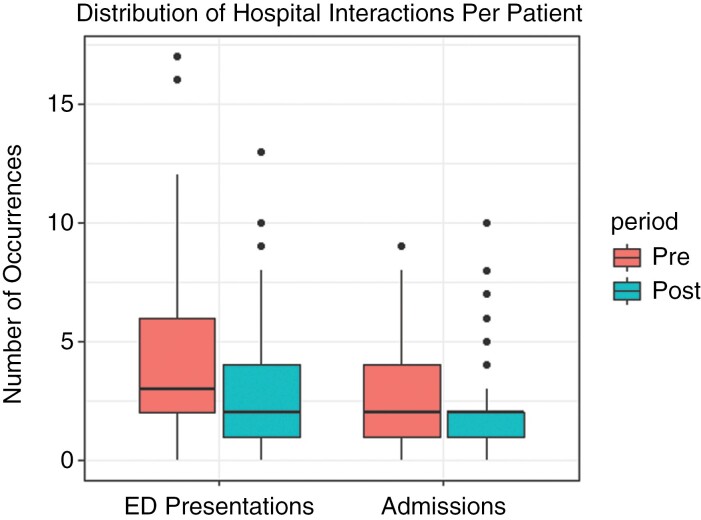
Frequency of ED presentations per patient between the 2 periods.

During the study period, there were a total of 701 presentations to the emergency department (ED) by the patients included in the analysis. Notably, there were 401 presentations prior to the introduction of BCCC, and 300 after the introduction of BCCC, with a trend towards patients having fewer overall ED presentations than prior to BCCC starting (Figure 2). Furthermore, there were 3 patients (3.3%) in the pre-BCCC group and 8 (8.3%) in the post-BCCC group who did not present to the ED at all during the study period.

A total of 427 (61%) ED presentations led to hospital admissions, 242 in the pre-BCCC group, and 185 in the post-BCCC group. The predominant category of admission was Acute Care in both groups. The 24% reduction in the total aggregate length of stay in hospital (1748 days vs 1332 days) reflects the reduction in the total number of hospital admissions between the pre and post periods. However, the median number of admissions were equal between the 2 groups (2.0). The reduction in the total number of hospital admissions in the post-BCCC translated into reduced length of stay for both acute care and palliative care admissions (Table 3).

**Table 3.** Hospital Resource Use Pre-post Comparison. Within Post-BCCC Cohort, Subgroups Were Stratified by Contact With the Program. All Monetary Figures Have Been Adjusted to 2019–20 Australian Dollars

	Pre	Post		
	*N* = 91	Overall, *N* = 96	No Contact, *N* = 54	BCCC Contact, *N* = 42
ED Visits				
Mean (Median)	4.4 (3.0)	3.1 (2.0)	2.7 (2.0)	3.6 (3.0)
Minimum:Maximum	0:17	0:13	0:9	0:13
Sum	401	300	148	152
Admissions				
Mean (Median)	2.7 (2.0)	1.9 (2.0)	1.7 (1.0)	2.2 (2.0)
Minimum:Maximum	0:9	0:10	0:8	0:10
Sum	242	185	94	91
Total Length of Stay				
Mean (Median)	19.2 (12.0)	13.9 (10.0)	13.2 (8.0)	14.7 (10.5)
Minimum:Maximum	0:214	0:69	0:52	0:69
Sum	1748	1332	715	617
Total ED Cost ($)				
Mean (Median)	3940 (3139)	2700 (2229)	2404 (2061)	3079 (2372)
Minimum:Maximum	0:13 034	0:9819	0:8136	0:9819
Sum	358 521	259 154	129 836	129 317
Total Admissions Cost ($)				
Mean (Median)	25 894 (20 359)	20 342 (17 998)	20 004 (12 536)	20 776 (20 457)
Minimum:Maximum	0:105 991	0:99 355	0:99 355	0:78 441
Sum	2 356 325	1 952 792	1 080 197	872 595
Total Cost ($)				
Mean (Median)	29 833 (23 479)	23 041 (19 838)	22 408 (13 429)	23 855 (23 407)
Minimum:Maximum	0:111 475	0:101 620	0:101 620	0:87 054
Sum	2 714 847	2,211 946	1 210 034	1 001 912

### Cost-Offset Analysis

**Figure 3.**
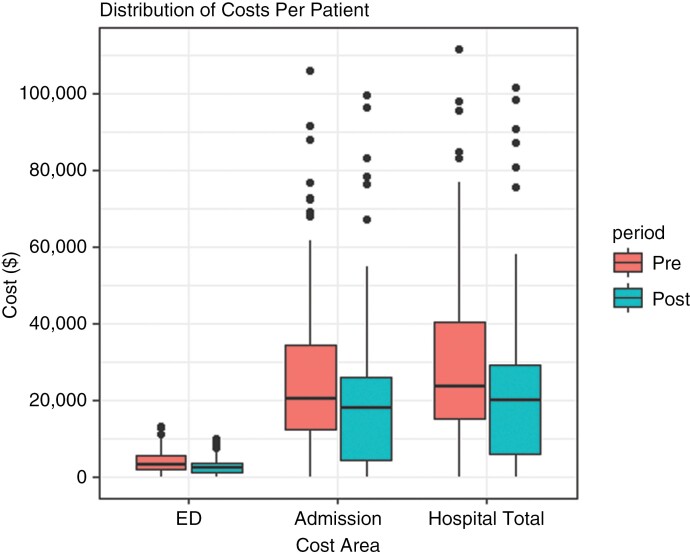
Costs per patient.

When the NEPs were adjusted for comparison, the mean cost of an emergency department attendance was calculated to be similar between comparison periods, after adjusting for price base differences. The mean cost of an inpatient visit increased by AUD$819 in real terms. None of the mean differences were statistically significant (Figure 3). However, analyses of these data showed a fall in the number of ED presentations that led to an inpatient admission and 25% fall in total length of stay. Hence, the average cost of treating a patient decreased post-BCCC, by AUD$1200 in ED and by AUD$5500 for inpatient admissions. Aggregating this per patient saving across the entire post-BCCC cohort translated to a total saving of AUD$502 901, or an annual saving of AUD$167 634.

Comparing this saving against the cost of employing the care coordinator, calculated as the wage cost plus on-costs, the program shows an annual net saving of AUD$4410.

Breaking down the post-BCCC cohort, a total of 44% of patients had contact with the BCCC program. On average, this subgroup had more ED visits (3.6 vs 2.7), and subsequent admissions (2.2 vs 1.7), and a longer length of stay (14.7 days vs 13.2 days) compared to those in the post-BCCC cohort who had no contact with the coordinator (Table 3). However, numerically the post-BCCC cohort in total has less ED presentations and admissions compared to the pre-BCCC cohort.

## Discussion

This is the first study looking at the objective treatment outcome and health utilization differences with or without the presence of cancer care coordinators. The study demonstrated that although survival of patients with glioblastoma were not improved by the BCCC, their access to acute hospital care reduced which translated into a reduction in utilization of scarce health resources. It is well established in literature that cancer care coordination improves on a variety of qualitative outcomes for patients, carers and clinicians involved.^4,9–11^ Cancer care is difficult for patients and their relatives who are often navigating the complex and compartmentalized health system, dealing with a life changing diagnosis, then also having to manage symptoms from the cancer and the treatments. Many studies looking at models of cancer care coordination looked at either areas of deficiency in the current models,^9,12^ health-related quality of life outcomes from cancer care coordination,^13^ and 1 study looked at cost-utility modeling in patients with colorectal cancer.^6^ It must be stressed that the BCCCs in this study are specialized nurses who offer not only care coordination but also clinical advice in consultation with the treating physicians.

Our within group analysis of the post-BCCC group identified that patients who had contact with the BCCC had more ED visits, admissions and longer length of stay. However, these findings do not undermine the aggregate cost reductions seen with the introduction of the BCCC as although all patients diagnosed with a primary brain tumor had contact with a BCCC, only those patients who had more severe symptoms would have contacted the BCCC for advice. Hence, variation within the post-BCCC cohort, combined with the pre-post comparison, suggest that the BCCC program reduced hospital demands of brain cancer patients with more severe symptoms.

One of the strengths of the study is that glioblastoma is a good platform to assess the effect of cancer care coordinators on the objective outcomes investigated in this study. The standard of care therapy was established in the seminal paper from Roger Stupp in 2005, which consists of concurrent chemoradiotherapy with temozolomide followed by adjuvant therapy with temozolomide alone.^3^ This treatment paradigm has not shifted in the 15 years since the study was published and when the last patient reviewed in this study was diagnosed. As seen in Table 1, there were no major changes in the treatment provided to patients during the study period with a similar proportion of patients having had gross resections then had the Stupp protocol. This is unlike other tumors, where there have been significant shifts in the treatment paradigm with improvements in treatment response and survival, and any potential differences seen in outcomes in the patients could, at least in part, be explained by these changes. Another strength of the study is the health economics analysis, which was not modeled but calculated from real world utilization data obtained from the medical records, thus providing more robust evidence of the cost improvements generated by the brain cancer coordinator.

Some of the limitations of the study are that this is a study looking at the impact of the rain Cancer Care Coordinator who are senior oncology nurses, and thus the outcomes seen in this study may not necessarily translate 1:1 to Cancer Care Coordinators in other tumor types. Furthermore, due to the nature of retrospective cohort studies, there were limitations due to missing data in some patients, especially regarding known predictive markers such as MGMT promoter methylation status and there were an uneven number of patients lost to follow-up between the 2 groups. In addition, the exact nature of the interactions between the BCCC and the patients and its influence were unable to be accurately investigated. The exact reason for why 56% of patients in the post-BCCC group did not have a recorded interaction with the BCCC is not clear. The data which was collected to analyze contact with BCCC was based on documented entries from the BCCC in the oncology EMR system. Certainly, there is a possibility that not all interactions were recorded and this may have led to this discrepancy due to documentation bias. However, given that the most common reason for contact with a BCCC was for medical advice, the patients interacting with the BCCC with documentation most likely had more severe symptoms, resulting in the ED visit and admission results seen in our research. Finally, another limitation of this study is that the health economics analysis was only available on patients who accessed the public health system, and did not include any patient contact, presentation to ED or admissions to the private hospitals. However, as seen in Table 1, in our data the number of patients who had treatment at the private hospital was small (19%), thus their contribution to the health economic analysis would likely to have been small. Furthermore, due to the structure of the public and private health systems in the Hunter New England region, most patients who needed hospital review and/or admissions due to illness would have utilized the public hospital systems despite undergoing treatment through the private system.

In conclusion, our study suggests that patients suffering from GBM with access to a Brain Cancer Care Coordinator are not likely to live longer, however they may present to hospital less often and may spend less time in acute care. This is likely to result in an improved quality of life and in view of the likely health resource savings it suggests that the BCCC are cost effective. This study objectively supports the ongoing presence of support of the Brain Cancer Care Coordinator in patient care.

## Supplementary material

Supplementary material is available online at *Neuro-Oncology* (https://academic.oup.com/neuro-oncology).

npae030_suppl_Supplementary_Table_S1

## References

[1] Australian Institute of Health and Welfare. Cancer in Australia 2021. Published online 2021. doi:10.25816/YE05-NM50

[2] Australian Institute of Health and Welfare. Brain and Other Central Nervous System Cancers.; 2017. Accessed February 24, 2023. https://www.aihw.gov.au/reports/cancer/brain-other-central-nervous-system-cancers/contents/table-of-contents

[3] Stupp R , Mason WP, van den Bent MJ, et al; European Organisation for Research and Treatment of Cancer Brain Tumor and Radiotherapy Groups. Radiotherapy plus concomitant and adjuvant temozolomide for glioblastoma. N Engl J Med. 2005;352(10):987–996. 15758009 10.1056/NEJMoa043330

[4] Tan AC , Ashley DM, López GY, et al. Management of glioblastoma: State of the art and future directions. CA Cancer J Clin. 2020;70(4):299–312. 32478924 10.3322/caac.21613

[5] Leone A , Colamaria A, Fochi NP, et al. Recurrent glioblastoma treatment: State of the art and future perspectives in the precision medicine Era. Biomedicines. 2022;10(8):1927. 36009473 10.3390/biomedicines10081927 PMC9405902

[6] Gorin SS , Haggstrom D, Han PKJ, et al. Cancer care coordination: A systematic review and meta-analysis of over 30 years of empirical studies. Ann Behav Med: Publ Soc Behav Med. 2017;51(4):532–546. 10.1007/s12160-017-9876-2 28685390

[7] Campbell C , Craig J, Eggert J, Bailey-Dorton C. Implementing and measuring the impact of patient navigation at a comprehensive community cancer center. Oncol Nurs Forum. 2010;37(1):61–68. 20044340 10.1188/10.ONF.61-68

[8] Koh C , Nelson JM, Cook PF. Evaluation of a patient navigation program. Clin J Oncol Nurs. 2011;15(1):41–48. 10.1188/11.CJON.41-48 21278040

[9] Singh-Carlson S , Wong F, Trevillion K, Reynolds G. Impact of a breast cancer care RN-coordinator program on patients’ distress level. Canadian Oncol Nurs J—Rev Can Nurs Oncol 2018;28(4):250–255. 10.5737/23688076284250255 PMC6516931 31148810

[10] Collett GK , Durcinoska I, Rankin NM, et al. Patients’ experience of lung cancer care coordination: a quantitative exploration. Support Care Cancer: Offic J Multinat Assoc Support Care Cancer. 2019;27(2):485–493. 10.1007/s00520-018-4338-3 29980906

[11] Blakely T , Collinson L, Kvizhinadze G, et al. Cancer care coordinators in stage III colon cancer: A cost-utility analysis. BMC Health Serv Res. 2015;15:306. 26238996 10.1186/s12913-015-0970-5 PMC4523949

[12] Temel JS , Greer JA, Muzikansky A, et al. Early palliative care for patients with metastatic non–small-cell lung cancer. N Engl J Med. 2010;363(8):733–742. 20818875 10.1056/NEJMoa1000678

[13] Harris PA , Taylor R, Thielke R, et al. Research electronic data capture (REDCap)—A metadata-driven methodology and workflow process for providing translational research informatics support. J Biomed Inform. 2009;42(2):377–381. 18929686 10.1016/j.jbi.2008.08.010 PMC2700030

[14] Harris PA , Taylor R, Minor BL, et al; REDCap Consortium. The REDCap consortium: Building an international community of software platform partners. J Biomed Inform. 2019;95:103208. 31078660 10.1016/j.jbi.2019.103208 PMC7254481

[15] Australian Institute of Health and Welfare. Hospital service—care type, code N[N]. Published March 4, 2019. Accessed February 22, 2023. https://meteor.aihw.gov.au/content/711010

