# Supplementary material for: The impact of brain cancer care coordinators on healthcare utilization and outcomes in patients with glioblastoma
Source: Neurooncol Pract. 2024 May 4;11(5):575–82. doi: 10.1093/nop/npae030 (PMC11398931; doi:10.1093/nop/npae030)
Supplement: npae030_suppl_Supplementary_Table_S1 [file npae030_suppl_supplementary_table_s1.docx]

Supplementary Table 1. Australian Institute of Health and Welfare Hospital service care type definitions relevant to study – online only

| Acute care | Acute care is care in which the primary clinical purpose or treatment goal is to:   - manage labour (obstetric) - cure illness or provide definitive treatment of injury - perform surgery - relieve symptoms of illness or injury (excluding palliative care) - reduce severity of an illness or injury - protect against exacerbation and/or complication of an illness and/or injury which could threaten life or normal function - perform diagnostic or therapeutic procedures. |
| --- | --- |
| Palliative care | Palliative care is care in which the primary clinical purpose or treatment goal is optimisation of the quality of life of a patient with an active and advanced life-limiting illness. The patient will have complex physical, psychosocial and/or spiritual needs. |
